# Sub-vastus approach versus the medial parapatellar approach in primary total knee: a randomised controlled trial [ISRCTN44544446]

**DOI:** 10.1186/1745-6215-7-23

**Published:** 2006-07-31

**Authors:** Stephen Bridgman, Gayle Walley, Gilbert MacKenzie, Darren Clement, David Griffiths, Nicola Maffulli

**Affiliations:** 1 University of Keele, Keele University Medical School, Thornburrow Drive, Hartshill, Stoke-on-Trent, Staffordshire, ST4 7QB, UK; 2 University Hospital of North Staffordshire NHS Trust, Newcastle Road, Stoke-on-Trent, ST4 6QG, UK; 3 Newcastle-under-Lyme Primary Care NHS Trust, Civic Offices, Merrial Street, Newcastle-under-Lyme, ST5 2AZ, UK; 4 University of Limerick, Dept. of Mathematics & Statistics, University of Limerick, Limerick, Ireland

## Abstract

**Background:**

Thirty thousand knee replacements are performed annually in the UK. There is uncertainty as to the best surgical approach to the knee joint for knee arthroplasty. We planned a randomised controlled trial to compare a standard medial parapatellar arthrotomy with sub-vastus arthrotomy for patients undergoing primary total knee arthroplasty in terms of short and long term knee function.

**Methods:**

Patients undergoing primary total knee arthroplasty at the local NHS Trust are to be recruited into the study. Patients are to be randomised into either the subvastus or medial parapatellar approache to knee arthroplasty. The primary outcome measures will be the American Knee Society and WOMAC Scores. The secondary outcome measures will be patient based measures of EuroQol and SF-36. All outcomes will be measured pre-operatively, 1, 6, 12 and 52 weeks post-operatively. We will also review pain intensity using a pain and analgesia diary. Ease of surgical exposure and complications will also be analysed.

**Discussion:**

Evidence is lacking concerning the best surgical approach to the knee joint for patients undergoing primary total knee replacement. This pragmatic randomised trial tests the hypothesis that the sub-vastus approach is significantly superior to the standard medial parapatellar approach in terms of short and long term knee function.

## Background

There is general agreement about the value of knee replacement surgery, with approximately 30,000 knee replacements performed annually in the UK. However, there is uncertainty as to the best surgical approach to the knee joint.

Total knee replacements through a standard medial parapatellar arthrotomy [1] are routinely carried out. The sub-vastus (southern) arthrotomy has been suggested as an alternative [2]. There have been two previous published trials of the sub-vastus approach versus a standard medial parapatellar approach.

In the first of these [3], 20 patients undergoing bilateral knee arthroplasty were studied, with follow-up at 1, 4 and 12 weeks post-operatively. The patients whose knee was approached by the sub-vastus method showed 35% and 16% greater quadriceps strength at 1 week and 1 month respectively. In addition, the authors highlighted several anatomical advantages. The approach maintains the medial parapatellar blood supply and preserves the extensor mechanism [4-6]. Thus, theoretically, it decreases the likelihood of patellar subluxation, fracture, or patellar avascular necrosis (reported as having an incidence of 17%, 21% and 10% respectively) [7,8].

In the second of these trials [4], 41 consecutive primary knee arthroplasties were studied with follow-up at 6 months. Patients who had the sub-vastus approach were able to straight leg raise 4 days sooner (p = 0.008) and used 40% less post-operative analgesia (p = 0.05). They also spent on average 3 days less in hospital (p = 0.01).

The subvastus approach has also been reported to have decreased wound complications, a shorter hospital stay, a reduction in analgesia usage and an early return to function when compared to the standard median parapatellar approach [2,4,9].

There were problems with the validity and quality of both studies, as they were not truly randomised, and involved only a small number of patients and follow-up was short. Long term follow-up is needed to determine whether potential short term benefits increase long term effectiveness. A larger sample size will allow a wider range of outcomes to be investigated (in particular knee function) and a broader range of patients to be recruited to improve external validity and allow for sub-group analysis (e.g. by age and sex). Furthermore, involving more surgeons will allow assessment of variation in outcome by individual surgeons.

We propose, therefore, a fully randomised controlled trial, with long-term follow-up, in a large sample population with patient-based outcomes to compare the standard approach with the sub-vastus approach in total knee replacement.

## Objectives

This trial proposes to:

1. study the effectiveness and efficiency at 1, 6, 12 and 52 weeks of a standard medial parapatellar arthrotomy compared with a sub-vastus arthrotomy in primary total knee replacement.

2. examine both clinical and economic outcomes of care of a standard medial parapatellar arthrotomy compared with a sub-vastus arthrotomy in primary total knee replacement at 1, 6,12 and 52 weeks.

3. examine patient based outcomes of care (EuroQol [10], WOMAC [11], pain (visual-analogue scale)) of a standard medial parapatellar arthrotomy compared with a sub-vastus arthrotomy in primary total knee replacement at 1, 6,12 and 52 weeks.

## Trial summary

**Figure 1:**
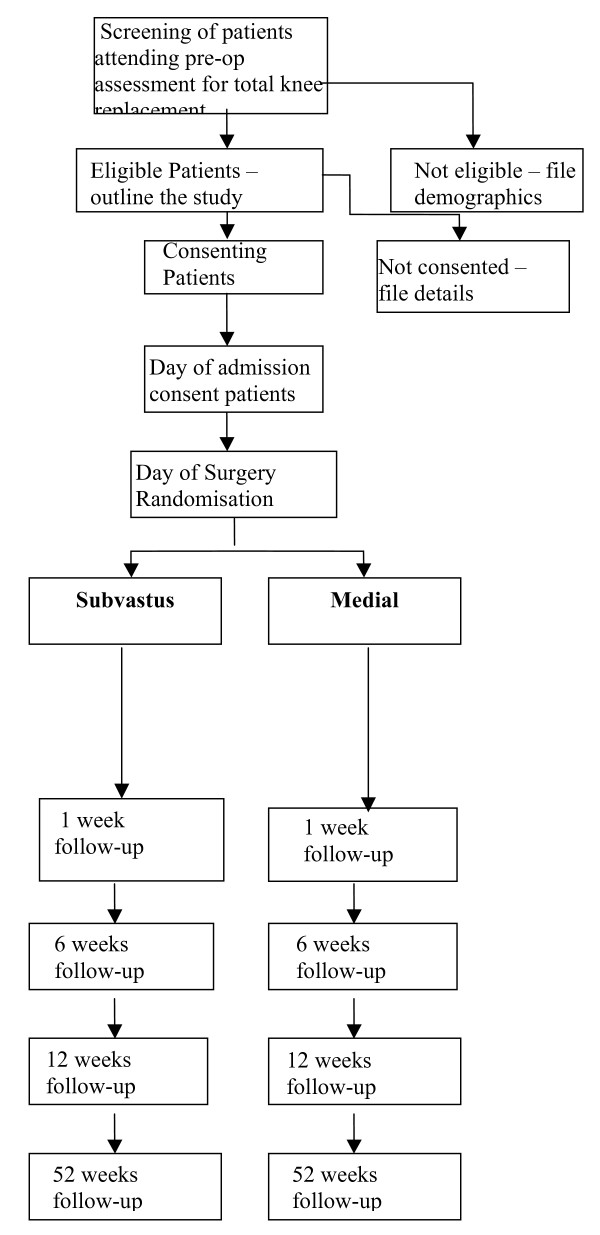
Flow Chart.

This study aims to compare the effectiveness and efficiency of a standard medial parapatellar approach versus a sub-vastus approach in total knee replacement and to evaluate the clinical outcomes of care in each group. We propose a randomised controlled trial, with follow-up at 52 weeks, in a large sample population of 230 patients with patient based outcomes. The trial summary can be seen in figure 1. Patients will be recruited at their pre-operative assessment with randomisation taking place on their day of admission for surgery. Clinical and functional assessments will be carried out by a research nurse and physiotherapist respectively, pre and post-operatively, with the physiotherapist unaware of the approach taken. The primary outcome measure for the study is the Knee Society Score [12] and this will be collected at baseline and at 1, 6, 12 and 52 weeks post-operatively. Other outcome measures will include patient based (EuroQol 5D [10], WOMAC Index [11]), clinical (complications, blood loss, use of analgesia) and functional (range of motion) measures. Data will be analysed on an intention to treat basis with the principal comparisons being the mean difference in the Knee Society Score between the two groups at 1, 6, 12 and 52 weeks post-operatively.

## Design

### Study type

Randomised controlled trial comparing standard medial parapatellar arthrotomy with a sub-vastus arthrotomy in total knee replacement.

### Participants

Participants will be patients undergoing primary bi (tibial-femoral joint) or tri (tibial-femoral-patellar) compartmental knee replacement for any indications. These patients will be recruited from North Staffordshire Hospital, Stoke on Trent.

## Treatment details

A Low Contact Stress knee prosthesis manufactured by Depuy will be used. Both consultant orthopaedic surgeons and consultant-supervised Orthopaedic Specialist Registrars will be eligible to perform each of the approaches.

### Skin incision

The prosthesis will be inserted via a mid-line skin incision beginning over the medial portion of the quadriceps tendon, four inches above the upper border of the patella. The incision will continue in a gentle curve following the medial margin of the quadriceps tendon, patella and patella tendon. It will then cross the upper end of the tibia and end inferior to the tubercule of the tibia. Exposure of the knee joint will then occur either by a standard medial parapatellar arthrotomy [1] or sub-vastus arthrotomy [2].

### Standard medial parapatellar approach

A straight incision will be made through the three layers of fascia over the quadriceps and the patella tendons. The synovia and deep aponeurosis will be divided medial to the patella and the quadriceps tendon will be separated in the line of its fibers just lateral to the insertion of the vastus medialis. The patella tendon will be freed along its medial border to the level of the tibial tubercule, exposing the infrapatellar bursa. The patellar will be dislocated laterally.

### Sub-vastus approach

Fascia layer I will be incised inline with the mid-line skin incision. At the level of the patella, it will be incised slightly medially. Beginning proximally, the fascia layer I will be raised off the perimuscular fascia of the vastus medialis down to its insertion site. The inferior edge of the vastus will be lifted off the periosteum and intermuscular septum with blunt finger dissection for approximately 10 cm proximal to the adductor tubercle. The tendinous insertion of the vastus medials will be incised about midpatella. Care will be taken not to incise the synovium. Lifting the extensor mechanism anterior and lateral will allow a curvilinear medial arthrotomy beginning in the suprapatellar pouch and ending at the tibial tubercule. Sharp soft tissue release from the proximal tibia will be performed. The patella will be everted laterally with the knee in full extension. The knee will be flexed while the vastus medialis will be bluntly dissected off intermuscular septum proximally. If a lateral release is needed, it will be carried out from the outside in, with the knee in full flexion.

## Eligibility

A patient will be eligible if:

(i) They require a bi or tri compartmental knee replacement

(ii) They require a unilateral knee replacement

(iii) They have given their informed consent

(iv) The surgeon has no clear preference for either of the approaches

A patient will be ineligible to enter the trial if:

(i) They need a revision knee replacement

(ii) They have had, or will require a major arthrotomy in the other knee in a 12 month period

(iii) They have had previous open surgery in or around the knee in the previous 12 months e.g. high tibial osteotomy, femoral osteotomy, ORIF for fracture, patellar realignment, patellectomy and open meniscectomy.

(iv) They require a bi-lateral knee replacement at a single visit

(v) They have a valgus angle of ≥ 20°

## Recruitment

Patients will be recruited over a 12 month period. Surgical lists will be reviewed weekly to identify all patients referred to the participating hospital centre and listed for total knee replacement. Their notes will be screened prior to their pre-operative outpatient appointment. At their pre-operative visit patients will be informed of the nature of the study by their consultant surgeon. Eligible patients will be provided with an information leaflet explaining the nature of the study to them. They will be invited to ask questions regarding the research. Patients will then be asked to return home and think about whether or not they wish to participate. During the 4–6 week period following their pre-operative visit and prior to admission for surgery, patients will be contacted by the research nurse to discuss their participation in the study and to address any unanswered questions they may have. On the day of admission, the patients will have a further opportunity to discuss participation with the research nurse involved in the trial. Those patients wishing to take part will be invited to sign an informed consent form by their surgeon. Following consent being obtained, patients will be randomised on their day of admission to either a sub-vastus or medial parapatellar approach.

## Randomisation

Patients will be randomised according to a minimisation method using a telephone randomisation service. The minimisation variables will be surgeon, and gender. Randomisation will take place after informed consent will have been given, eligibility criteria will have been checked, and baseline measurements will have been taken. The randomisation will take place on the day of admission. This will be carried out at the co-ordinating centre. At this telephone call, basic descriptive information will be given (patient's name, sex, date of birth, hospital and surgeon). After this phone call, the patient will be considered irrevocably to have entered in the trial for the purposes of the research.

## Assessment of outcome

The primary outcome measure for this study will be the Knee Society Score [12] at 52 weeks, and will be carried out by a trained independent assessor (physiotherapist) who will be blinded to the surgical approach taken.

### Secondary outcomes – patient based

1. EuroQol-5D [10]. A simple, generic measure of health outcome. It allows an accurate self-description of current health related quality of life, assessing mobility, self-care, usual activity level, pain/discomfort and anxiety/depression.

2. WOMAC Osteoarthritis Index [11]. An osteoarthritis specific measure of health outcome, assessing pain, stiffness and activity level.

3. SF-36 [13]. A comprehensive generic measure of health outcome.

4. Time to normal activities/return to function (days/weeks)

5. Pain (7 day pain diary – visual analogue scale 0–100)

### Secondary outcomes – medical records

1. Complications (in hospital or requiring readmission during 12 month follow-up)

2. Length of stay in hospital (days)

3. Tourniquet time (minutes)

4. Blood loss (litres)

5. Post-operative use of analgesia (7 day analgesia diary recorded by nurse)

6. Surgeons ease of exposure score (VAS 0–100) – form inserted in notes

### Secondary outcomes – independently assessed by physiotherapist

1. Flexion/extension range of motion (degrees)

2. Time to straight leg raise (days)

### Secondary outcomes – health economic

1. Cost of inpatient care including operative/diagnostic, length of stay, length of operation staff costs, complications/re-admissions and consumables

## Assessments/data collection

All trial proformas will be supplied in a separate booklet and will form the basis of the patients case report form. Assessment will be conducted pre, peri and post-operatively. Data on the primary and all patient based and independently assessed secondary outcome measures will be collected at 1, 6, 12, and 52 weeks postoperatively. These assessments are timed to coincide with routine clinical practice.

## Statistical considerations

### Sample size

In a group of 86 patients undergoing total knee arthroplasty the mean 1 year post-operative Knee Society Score was 172.2 with a standard deviation of 20 and a range of 98–200 [14]. To detect a 5% increase in the post-operative Knee Society Score in the sub-vastus group compared to the medial parapatellar group with a two sided significance level of 5% and a power of 90% a sample size of 105 patients would be required in each group. An additional 10% will be recruited to each group to allow for any patients lost to follow-up, giving a total sample size of 231.

### Data analysis

Intention to treat analysis – all patients entered into the trial will be followed up in the group to which they were randomly allocated regardless of which treatment they received. Continuous data will be analysed using repeated measures analysis of variance while the chi square test will be used to compare all categorical data. Baseline characteristics of the group will be compared using appropriate parametric and non parametric tests to ensure balance has been achieved with randomisation.

The principal comparisons will be between:

a) Comparison of the mean total Knee Society Score between the two groups at 1, 6, 12 and 52 weeks post-operatively.

b) Comparison of all secondary outcome measures between the two groups.

### Protocol deviations

Any deviation from the agreed protocol will be recorded. Any amendments that may be required once the trial has been initiated will be submitted for ethics committee approval prior to implementation.

### Interim analyses and Data Monitoring Committee (DMC)

The first monitoring analysis of the primary end will be performed once 50 patients have completed their 1 week post-operative assessment. The data will be supplied to an independent data monitoring committee (DMC), which will be asked to give advice on whether the accumulated data from this trial, justifies its continuation. The independent DMC will comprise a statistician, an orthopaedic surgeon, and a clinical researcher.

The decision to discontinue will only be made if the results are likely to be convincing to a broad range of clinicians including participants in the trial and the general clinical community. A nominal significance level for stopping the trial will be p <= 0.02 [15].

## Ethical considerations

This study has obtained ethical approval from the North Staffordshire Local Research Ethics Committee and Scientific Merit Committee.

## Informed consent

All eligible patients who agree to take part will sign an informed consent form. They will confirm that they have been given the information they require and that the study has been explained to them. Once a patient has consented to enter the trial, their GPs will be informed of their patients' involvement in the study.

## Confidentiality

Only the trial organisers/administrators will have access to patients' notes and questionnaires. All recorded information will be stored separately from patient names and addresses.

## Trial organisational structure

### Trial co-ordination

The day to day management of the trial will be co-ordinated from the local Musculoskeletal Research Department. The co-ordinating centre will be responsible for:

a) Processing and analysis of the trial data

b) Monitoring of data collection

c) Ensure confidentiality and security of all trial forms and data

d) Conduct data checking and cleaning

e) Interim and main analysis

### Clinical co-ordination

At the start of the trial representatives from the clinical centre along with the trial co-ordinator will visit surgeons with the aim of getting commitment to recruit patients into the study.

The Clinical Centre will:

(i) establish the trial locally with the assistance of the trial co-ordinator (for example getting agreement from colleagues, facilitating local research ethics committee approval, ensuring all clinical staff involved in the care of the patients are informed about the trial)

(ii) take responsibility for clinical aspects of the trial locally

(iii) be represented at SMAK arthroplasty trial meetings

A part-time study nurse will be based at the collaborating centre and will have responsibility for:

(i) keeping local staff informed about the trial and its progress

(ii) keeping regular contact with local surgeons and co-ordinating centre

(iii) identifying potential participants in advance of admission

(iv) taking responsibility for ensuring complete data collection

(v) getting arrangements in place for formal trial entry

(vi) facilitating progress of patients through the trial

In addition, a part time research physiotherapist will be based at the collaborating centre and will have responsibility for:

(i) assessment of functional capacity

(ii) liasing with research nurse to ensure timely assessment of outcome

## Discussion

There have been several trials comparing the subvastus and medial parapatellar approaches for patients undergoing primary total knee arthotomy. The major criticisms of the sub-vastus approach are poor and unpredicatable exposure, and difficulty with eversion of the patella [16,17]. Some studies have shown significant advantages in using the subvastus approach in relation to improved quadriceps strength [18], earlier straight leg raise, and lower analgesia use in the first week for the subvastus group [19,20]. These studies, however, only had small numbers and a short follow-up period, and not all patients were blinded to the approach used.

We present the rationale and design of a pragmatic randomised controlled trial comparing the sub-vastus approach to the standard medial parapatellar approach in terms of short and long term knee function for patients undergoing primary total knee replacement in which the patients are blinded to the approach used. The results of this trial will be beneficial for Orthopaedic Surgeons whom are under constant pressure to undertake techniques which aid quicker postoperative rehabilitation, and shorter hospital stay. The results of this trial will be published as soon as they become available.

## Competing interests

The author(s) declare that they have no competing interests.

## Authors' contributions

SB, DC and DG conceived the trial design. SB, NM and GW have been involved in subsequent adaptations and drafted the manuscript. DG, DC and GM have contributed to adaptations from the original design. All authors read and approved the manuscript.
